# Surgical treatment of aspergillosis by video-assisted thoracoscopy—challenging but effective and safe minimally invasive approach

**DOI:** 10.1093/icvts/ivad056

**Published:** 2023-05-02

**Authors:** Cezary Piwkowski, Piotr Skrzypczak

**Affiliations:** Department of Thoracic Surgery, Poznan University of Medical Sciences, Poznań, Poland; Department of Thoracic Surgery, Poznan University of Medical Sciences, Poznań, Poland

**Keywords:** Aspergillosis, Chronic pulmonary aspergillosis, VATS, Video-assisted thoracoscopy, Uniportal

The treatment of chronic pulmonary aspergillosis (CPA) is a major clinical challenge. It often includes the long treatment process, in usually immunocompromised patients, with multiple comorbidities or damaged lungs after previous tuberculosis or other causes [1]. Some of these patients require surgical treatment due to complications of CPA, with the most common: the severe haemoptysis or massive bleeding from the respiratory tract [2].

Surgical resection of aspergilloma is a definitive treatment option for patients with adequate pulmonary function [2]. The procedure's success depends on the complete resection of the aspergilloma without spillage of fungal elements into the pleural space [1–3]. Surgical treatment includes the radical removal of the aspergilloma, which could require an anatomical resection of the lung parenchyma, most often a lobectomy [4, 5].

Unfortunately, these procedures are usually technically demanding, mainly due to difficult operating conditions: massive pleural adhesions or damaged lung parenchyma [3]. This often makes it challenging to evenly locate the pulmonary vessels in the lung hilum during the operation. In addition, inflamed and calcified lymph nodes in the hilum, usually closely adjacent to the pulmonary artery branches, cause significant difficulties in safe dissecting and stapling.

Furthermore, complications after surgery due to CPA are relatively common. The most frequent are: persistent air leaks, residual pleural space, empyema, pneumonia, wound infection, bronchopleural fistula, respiratory failure, massive bleeding and death [1, 3]. For these reasons, selecting patients with CPA for surgical treatment should be meticulous, considering all potential risk factors.

Over the past 20 years, we have witnessed a dynamic development of minimally invasive techniques in thoracic surgery. Currently, in the leading thoracic surgery centres, most procedures are performed using the video-assisted thoracoscopy (VATS) method, including anatomical resections due to non-small-cell lung cancer [6].

Regardless of the type of VATS access and the number of surgical ports used, this method significantly reduces the surgical trauma associated with the surgical procedure compared to traditional thoracotomy [7]. The use of the VATS is associated with the following benefits for the patient: reduction of postoperative pain, lower number of postoperative complications, shorter hospital stay and faster recovery [8]. Most published studies evaluating the effectiveness of the VATS concern oncological patients operated on due to lung cancer. Therefore, assessing the efficacy and safety of VATS resections in patients with CPA is of great value. The literature still lacks available studies evaluating this method's effectiveness and safety based on many patients.

In the paper by Jiang *et al.* [9], the authors analysed the efficacy of surgery in the group of 348 patients with CPA. The study focused on comparing the treatment results of patients operated on due to CPA using the s-VATS and m-VATS. In the whole group, almost two-thirds (208 patients) were operated on using the minimally invasive VATS approach with multiport (m-VATS) or single port (s-VATS) access. After performing the propensity score matching analysis, the authors compared the 2 groups of 63 patients each. No significant differences among the groups were noticed in the operation time, blood loss, drainage duration, drainage volume and postoperative in-hospital stay. There was no 30-day postoperative death in any group.

However, in patients receiving lobectomy by s-VATS, the authors observed a shorter postoperative hospital stay than those who received lobectomy by m-VATS. In addition, patients in the s-VATS group had lower postoperative morbidity, had significantly lower pain intensity after surgery and experienced fewer analgesic days in 30-day follow-up than in the m-VATS group.

The very low percentage of conversions to thoracotomy in both groups is worth emphasizing. This proves the high quality of the surgical technique, extensive experience in performing minimally invasive procedures, regardless of the surgical access used, and an accurate qualification for the VATS procedures.

The article includes a long-term analysis of the treatment results of patients operated on in the years 2007–2019. However, the first study describing the uniportal surgical technique in the case of anatomical resections was published in 2011. Therefore, it should be assumed that in the early stage of the study, the m-VATS was the only available surgical procedure [10, 11]. More experience in the m-VATS technique was certainly gained during this long period, which could have influenced the study results. We also do not have information on the analgesic treatment protocol in the study material, which probably also evolved over the 13 years. Considering these limitations, we should cautiously evaluate the described results, especially those regarding hospitalization time or surgical trauma.

A related point to consider are previous studies comparing the postoperative course in patients after lobectomy performed with the VATS and thoracotomy [7]. According to one of the few randomized studies comparing VATS lobectomy versus conventional open lobectomy for lung cancer, the VIOLET study [7], it was found that VATS was associated with better recovery of physical function after 5 weeks compared with open surgery. However, in the longer follow-up, after 52 weeks, there were no differences in physical function nor in overall survival [7].

In conclusion, the most excellent value of this study is to prove the effectiveness and safety of the VATS in the surgical treatment of CPA in a large group of patients. The low number of complications and the low rate of conversion to thoracotomy evidence this. This method is equally effective and safe, regardless of the amount of ports used. Finally, obtaining similar results requires extensive experience in performing lung resection procedures using the VATS, not to mention the appropriate qualification of patients.
